# Genomic survey of *Clostridium difficile* reservoirs in the East of England implicates environmental contamination of wastewater treatment plants by clinical lineages

**DOI:** 10.1099/mgen.0.000162

**Published:** 2018-03-02

**Authors:** Danesh Moradigaravand, Theodore Gouliouris, Catherine Ludden, Sandra Reuter, Dorota Jamrozy, Beth Blane, Plamena Naydenova, Kim Judge, Sani H. Aliyu, Nazreen F. Hadjirin, Mark A. Holmes, Estée Török, Nicholas M. Brown, Julian Parkhill, Sharon Peacock

**Affiliations:** ^1^​ Wellcome Trust Sanger Institute, Hinxton, UK; ^2^​ University of Cambridge, Cambridge, UK; ^3^​ London School of Hygiene and Tropical Medicine, London, UK; ^4^​ University of Freiburg, Freiburg im Breisgau, Germany

**Keywords:** *Clostridium difficile*, genomic epidemiology, One Health, wastewater treatment plants

## Abstract

There is growing evidence that patients with *Clostridiumdifficile*-associated diarrhoea often acquire their infecting strain before hospital admission. Wastewater is known to be a potential source of surface water that is contaminated with *C. difficile* spores. Here, we describe a study that used genome sequencing to compare *C. difficile* isolated from multiple wastewater treatment plants across the East of England and from patients with clinical disease at a major hospital in the same region. We confirmed that *C. difficile* from 65 patients were highly diverse and that most cases were not linked to other active cases in the hospital. In total, 186 *C. difficile* isolates were isolated from effluent water obtained from 18 municipal treatment plants at the point of release into the environment. Whole genome comparisons of clinical and environmental isolates demonstrated highly related populations, and confirmed extensive release of toxigenic *C. difficile* into surface waters. An analysis based on multilocus sequence types (STs) identified 19 distinct STs in the clinical collection and 38 STs in the wastewater collection, with 13 of 44 STs common to both clinical and wastewater collections. Furthermore, we identified five pairs of highly similar isolates (≤2 SNPs different in the core genome) in clinical and wastewater collections. Strategies to control community acquisition should consider the need for bacterial control of treated wastewater.

## Data Summary

Sequence data deposited in the European Nucleotide Archive (ENA) (www.ebi.ac.uk/ena) under the individual accession numbers, along with the metadata of each isolate, are described in Table S1 (available in the online version of this article). There were restrictions on providing exact geographical locations of wastewater treatment plants. We confirm that all supporting data, code and protocols have been provided within the article or through supplementary data files.

**Impact Statement**

There is growing evidence that patients with *Clostridium difficile*-associated diarrhoea often acquire their infecting strain before hospital admission. To understand potential environmental reservoirs of *C. difficile*, we recovered *C. difficile* from wastewater treatment plants across a wide geographical area and used whole genome sequencing to reveal that strains within each wastewater treatment plant not only are highly diverse but also that some are toxigenic. Furthermore, we showed that some clinical strains are closely related to wastewater isolates. These results demonstrate the clinical significance of *C. difficile* in wastewater treatment plants in controlling the spread of *C. difficile*.

## Introduction

*Clostridium difficile* is a major cause of healthcare-associated infection worldwide [1–6]. Infection has been linked to nosocomial acquisition and epidemic spread of *C. difficile* [7], which has led to extensive hospital infection control measures combined with antimicrobial stewardship [8]. In the UK, there has been a major decline in incidence associated with a dramatic reduction in fluoroquinolone-resistant epidemic strains such as the hypervirulent ST1/ribotype 027 (027) [9]. In parallel with this has been a shift towards increasing community-acquired disease, which accounts for about one-third of cases and an increase in *C. difficile* ribotype diversity [10]. Recent studies in the UK employing whole genome sequencing have found that less than half of isolates causing *C. difficile* infection are genetically linked to isolates from active cases [11, 12].

Taken together, these observations suggest that patients with *C. difficile*-associated diarrhoea increasingly acquire their infecting strain prior to hospital admission. These marked changes in clinical and bacterial epidemiology necessitate consideration of alternative reservoirs for *C. difficile* [13–17]. Wastewater treatment plants have been proposed as potential reservoirs for *C. difficile*, and *C. difficile* ribotypes associated with clinical disease have been isolated previously from such sources [18–20], but surveillance of wastewater treatment plants and clinical reservoirs in the same geographical region using whole genome sequencing is lacking. Here, we investigate *C. difficile* in multiple wastewater treatment plants across the East of England, half of which were located downstream of hospitals, and compare these to clinical isolates from the same geographical region but sampled in a different time interval.

## Methods

### Isolates from patients with *C. difficile*-associated diarrhoea

A retrospective study was performed at the Cambridge University Hospitals NHS Foundation Trust between October 2012 and April 2013. Stool samples were processed at the on-site Public Health England Clinical Microbiology and Public Health Laboratory. Samples were tested using a commercial assay (*C. DIFF QUIK CHEK COMPLETE*; Techlab), which detects glutamate dehydrogenase (GDH) antigen and toxins A and B of *C. difficile*. Positive samples were cultured anaerobically for 48 h onto Brazier’s cycloserine-cefoxitin-egg yolk (CCEY) agar (BioConnections) using alcohol shock (https://www.gov.uk/government/publications/smi-b-10-processing-of-faeces-for-clostridium-difficile) and colonies with typical appearance were identified by MALDI-TOF MS (Biotyper version 3.1; Bruker Daltonics). Stool samples from which *C. difficile* was isolated were stored at −80 °C prior to repeated recovery of *C. difficile*, DNA extraction and sequencing. Clinical data were recorded on patient age, gender, date of hospital admission, discharge and ward movement, and previous admission to hospital in the preceding 6 months. Recurrent *C. difficile* infection was defined as the onset of diarrhoea 4 weeks or more after the end of the prior episode.

### Isolation of *C. difficile* from wastewater treatment plants

A cross-sectional study was performed in which 20 municipal wastewater treatment plants were sampled in the East of England between June 2014 and January 2015. Ten plants were situated immediately downstream of acute NHS Hospital Trusts and ten plants were not connected to acute hospital effluent. Paired samples of untreated (at inlet after initial screening to remove large solids but prior to reaching the primary settlement tanks) and treated (effluent at the point of release) wastewater were obtained from each plant. The main septic tank at the Cambridge University Hospitals NHS Foundation Trust was also sampled in September 2014. At each wastewater sampling point, two consecutive grab samples of 0.5 litres each were collected and mixed into 1 litre sterile bottles containing 18 mg sodium thiosulphate (Sigma-Aldrich). Triplicate neat [1, 10 and 100 ml (the last only for treated)] and dilutions of untreted (1 ml of 10^−1^ and 10^−2^) and treated (1 ml of 10^−1^) wastewater samples were concentrated using the filtration technique onto 0.45 µm pore size, mixed cellulose ester filter membranes (S-Pak; Merck Millipore). For wastewater plants 12–20, an alcohol shock step was additionally used on 1 and 10 ml neat samples (addition of 1 : 1 volume of 100 % ethanol for 30 min) prior to filtration to remove false-positive anaerobic vegetative growth. Membranes were placed aseptically onto chromID *C. difficile* selective plates (bioMérieux) and incubated anaerobically for 48 h at 37 °C. Multiple colonies with morphology consistent with *C. difficile* were picked from each positive plate and identified to species level by MALDI-TOF MS.

### DNA sequencing, genome assembly and population genomics analyses

DNA libraries were prepared according to the Illumina protocol and sequenced on an Illumina HiSeq2000 device with 100-cycle paired-end runs to give an average depth of 85-fold. Taxonomic identity was assigned to all short reads and assemblies using Kraken [21]. Multilocus sequence types (STs) were derived from Illumina read data using an in-house tool, available at https://github.com/sanger-pathogens/mlst_check. *De novo* multi-contig draft assemblies were produced using an improved algorithm based on Velvet [22]. The assembly pipeline gave an average total length of 4 216 955 bp (range 3 839 400–5 306 342) from 45 contigs (range 22–1866) with an average contig length of 119 057 bp (range 2843–187 572 bp) and an average N50, which is a statistic measured such that at least half of the nucleotides in the assembly belongs to contigs with the length equal to or longer than the statistic, of 432337.9 (range 140 791–841 201). The *de novo* assemblies were annotated using Prokka [23], and these were then used in the pan-genome analysis using Roary [24]. A cut-off of 95 % was used to define the locus match in the pan-genome analysis. Scoary [25], with 50 permutation tests for the empirical *P*-value, was used to identify accessory genes in isolates from wastewater versus clinical origin. After reconstruction of the core genome alignment, SNPs in the core genome were extracted using an in-house tool, available at www.github.com/sanger-pathogens/snp-sites. The SNP alignment was used to build a neighbour-joining tree with the ape package in R. The SNP alignment was also used to estimate a maximum-likelihood tree with FastTree 2.1.3 using the generalized time-reversible model. Phylogenetic trees aligned with the metadata and geographical data were visualized using Microreact (www.microreact.org), iTOL [16], FigTree (www.tree.bio.ed.ac.uk/software/figtree/) and Easyfig [17].

### Bayesian analysis

Putative sites of recombination (regions of high SNP density) were removed using Gubbins [26] with five iterations. The program beast v1.7 [27] was then applied to the SNP alignment. Various models were tested, including a strict molecular clock with uniform and lognormal distribution for base frequency and a General Time Reversible (GTR) model with a gamma correction for among-size variation. We executed three independent chains for 50 million generations with sampling every 10 generations and checked the convergence by considering an effective sample size of greater than 200, after leaving out the first 50 million initial states as the burn-in phase, using the Tracer software in the beast package. TreeAnnotator software, which is part of the beast package, was then used to merge the simulated tree to estimate the Bayesian tree. The key parameters such as root age and substitution rate from different models were in agreement and therefore the choice of prior model did not seem to change the results. We presented the results for the strict clock model.

The likelihood of the ancestral state was estimated for the presence and absence of toxin genes using the rerootingMethod function (the SYM method) in the phytools package in R. The tree was visualized using the plotTree function in the same package.

### Antimicrobial resistance determinants, virulence factors and plasmids

Putative virulence factors and resistance genes were identified using the srst2 package [28], and a 90 % coverage and similarity cut-off. The virulence factors database was obtained from (http://www.mgc.ac.cn/VFs/), and the resistance gene database was obtained from the srst2 package. The genomic regions encoding toxins A and B (PaLoc) and the binary toxin (CdtLoc) were initially identified using an in-house *in silico* PCR tool. The regions were then extracted and MUMmer [29] was used to map the whole genome assemblies to the identified genomic regions that contained the toxin genes. Except for two isolates where srst2 did not detect the toxin gene correctly, the results of the two methods were in agreement. Moreover, we ran blast on the elements against the non-redundant nucleotide NCBI database to annotate the genomic regions surrounding the genomic loci of toxin genes.

## Results

### Genomic epidemiology of *C. difficile* associated with disease and from wastewater treatment plants

**Fig. 1.**
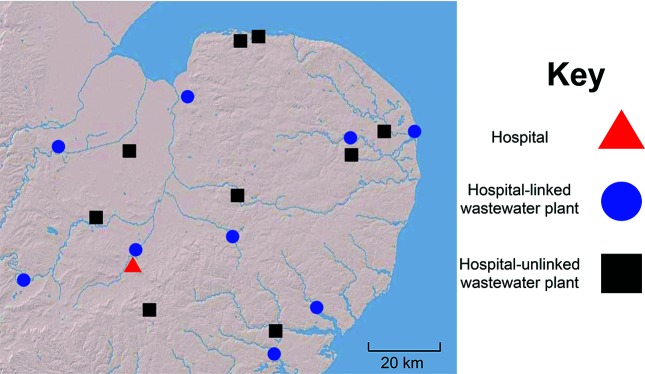
Location of hospital and wastewater treatment plants in the East of England where *C. difficile* isolates were recovered. Triangle, Cambridge University Hospitals NHS Foundation Trust. Wastewater treatment plans in direct receipt of hospital effluent (circle) or not in direct receipt of hospital effluent (square).

We defined the genomic epidemiology of *C. difficile* associated with human disease in our region through a retrospective observational study of 65 consecutive cases of *C. difficile*-associated diarrhoea over 8 months at the Cambridge University Hospitals NHS Foundation Trust, a 1100-bed acute hospital in the East of England. Four patients had more than one episode over the study period (three patients with two episodes, and one patient with three episodes, i.e. recurrent infections as defined in the Methods). A total of 70 clinical isolates were analysed. Furthermore, we examined *C. difficile* in hospital sewage and municipal wastewater treatment plants, with a particular focus on treated water released into the environment. A cross-sectional survey was conducted of 20 municipal wastewater treatment plants in the East of England (10 situated immediately downstream of acute NHS Hospital Trusts and 10 not directly connected to acute hospital effluent). Paired samples of untreated and treated wastewater were cultured from each plant. A sample from the main septic tank at the Cambridge University Hospitals NHS Foundation Trust was also cultured. *C. difficile* was isolated from the hospital sewer and from 18 treatment plants, nine of which did not directly receive hospital waste (Fig. 1). *C. difficile* was isolated from treated wastewater at the point of release onto surface waters (rivers, canals or the sea) from all 18 plants. A total of 186 colonies (hereafter termed isolates) were picked from primary wastewater culture plates and sequenced, with greater emphasis placed on treated wastewater (135/186, 73 %). The median (range) number of isolates sequenced per treatment plant was 9.8 (1–21), and 97/186 (52 %) isolates were from plants in direct receipt of hospital waste.

**Fig. 2.**
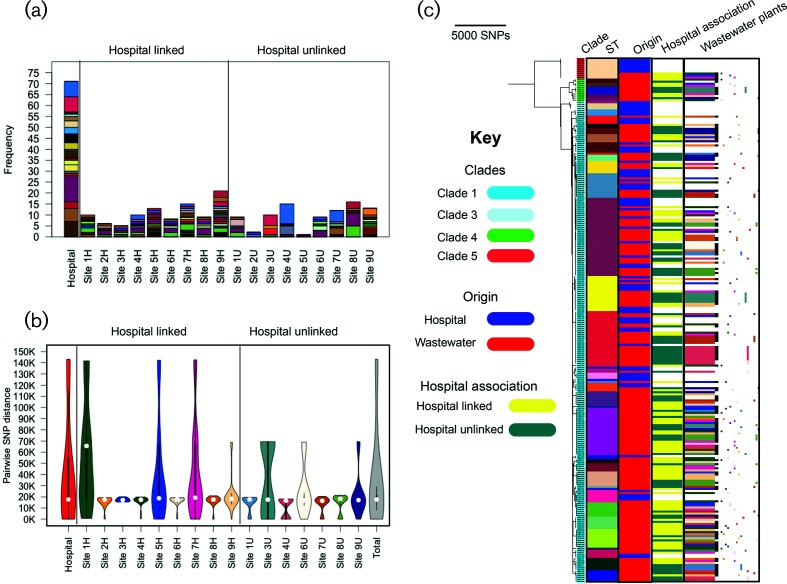
Phylogenetic analysis of *C. difficile* isolates from patients and wastewater. (a) Multilocus STs for 70 clinical and 186 wastewater isolates. Clinical isolate STs are shown in a single bar, while wastewater STs are shown according to treatment plant. Each colour refers to one ST. (b) Pairwise core genome SNP distance for clinical isolates and for isolates from each treatment plant. The total histogram shows the pairwise SNP distance for the whole population. Boxes denote the interquartile range and whiskers indicate the boundary of 1.5 times the interquartile range. The white marker shows the median value. The coloured area is the probability density of the data at different values. (c) Neighbour-joining phylogenetic tree for 256 isolate genomes and the distribution of clinical and wastewater isolates. These are labelled by clade, ST, origin, link to hospital waste (only for wastewater isolates) and wastewater plants. The black bars in the wastewater plants column show the post-treatment isolates. The circle and square signs show hospital linked and hospital unlinked isolates within wastewater treatment plans, respectively. Each color corresponds to one plant. The full and empty signs in the same color show the post- and pre-treatment isolates, respectively, for each plant.

Initial analysis based on multilocus STs revealed 19 distinct STs in the clinical collection and 38 STs in the wastewater collection (Fig. 2a). Isolate diversity was also observed in the majority of individual treatment plants, with a median (range) number of STs per plant of 6.5 (1–13) (Fig. 2a, c). Comparison of STs in clinical versus wastewater collections revealed that 13 out of 44 STs were common to both (Table S1).

*C. difficile* consists of six different clades termed 1–5 and C-I, of which clade 2 has been characterized as being associated with more severe disease (hypervirulent) [30]. A phylogenetic analysis based on 112378 SNPs in the core genomes of the 70 clinical and 186 environmental isolates showed that our collection lacked isolates belonging to clade 2 (consistent with a previous study of clinical isolates from across Europe [31]) and clade C-I, but included isolates belonging to the other major *C. difficile* clades. The majority of isolates belonged to clade 1 (231/256), the remainder being distributed across other clades (4/256 clade 3, 11/256 clade 4 and 10/256 clade 5) [30] (Fig. 2c). Clade 4 was entirely composed of environmental isolates, two of which were assigned to ST37.

The phylogenetic tree demonstrated extensive mixing between the two groups (Fig. 2c). Genetic relatedness of the 70 clinical isolates was defined by a pairwise comparison of core genomes to identify the difference in SNPs between each pair. Isolates were classified as genetically related if they were <2 SNPs different, genetically distinct if >10 SNPs different and indeterminate if 3–10 SNPs different, as previously described [11]. This analysis showed that 26 isolates were genetically related to at least one other isolate, five isolates were indeterminate and 39 isolates were genetically distinct (median 6673 SNPs, range 11–65 104). A phylogenetic tree based on SNPs in the core genome of the 70 isolates demonstrated considerable diversity overall (Fig. S1). Analysis of the genetically related isolates confirmed that two of four patients with recurrence had infectious episodes caused by the same strain. Epidemiological analysis of patients associated with the 26 genetically related isolates revealed 16 transmission events, of which seven were associated with direct ward contact, five with indirect ward contact and four with hospital-wide contact. These data confirm that the majority of *C. difficile*-associated diarrhoea was unlinked to other active cases in the hospital, suggesting that the patient had acquired the infecting organism prior to admission or from unrecognized cases in the hospital.

On pairwise core genome comparison, the most closely related clinical–wastewater isolate pair differed by 1 SNP for treatment plants that directly received hospital effluent and 4 SNPs for plants that did not. Within the wastewater collection, we identified 26 pairs of closely related isolates (<2 SNPs difference) in which each isolate of the pair was from a different treatment plant (indicative of dissemination of the same strain), together with 92 pairs of highly similar isolates (<2 SNPs difference) in which each isolate in the pair was from the same plant (indicative of multiple colony picks of the same strain). Isolate relatedness based on place of origin was identified through a network analysis (Fig. S2). This relatedness was nested within a context of genetic diversity, in which the mean pairwise core genome SNP distance of isolates cultured from 15 individual plants was largely similar (~20 000 SNPs) to that of the entire clinical and wastewater collections (Fig. 2b).

ST11 (ribotype 078) has been recovered from animals and wastewater and recognized over the last decade as a pathogenic strain associated with community- and hospital-acquired disease in Europe [10, 18, 32–34]. In light of this, we used Bayesian analysis to compare the 10 ST11 isolates in our collection (seven clinical isolates, three wastewater isolates each from a different plant in receipt of hospital effluent) with the genomes of 67 ST11 isolates recovered in the Netherlands from hospitals and pig farms (Table S2), where transmission between farmers and pigs has been observed [33]. This demonstrated recent mixing of isolates in our collection with isolates from animals, as well as healthy and diseased humans in the Netherlands, with four clades diverging within the past 20 years (Fig. S3).

### Distribution of toxin genes in *C. difficile* associated with disease and from wastewater treatment plants

Having established that wastewater treatment plants in the East of England release *C. difficile* into the environment, we used whole genome sequencing to evaluate the clinical relevance of isolates destined for release. This analysis considered the presence of genes encoding putative virulence determinants (primarily toxin genes) and antibiotic resistance determinants.

**Fig. 3.**
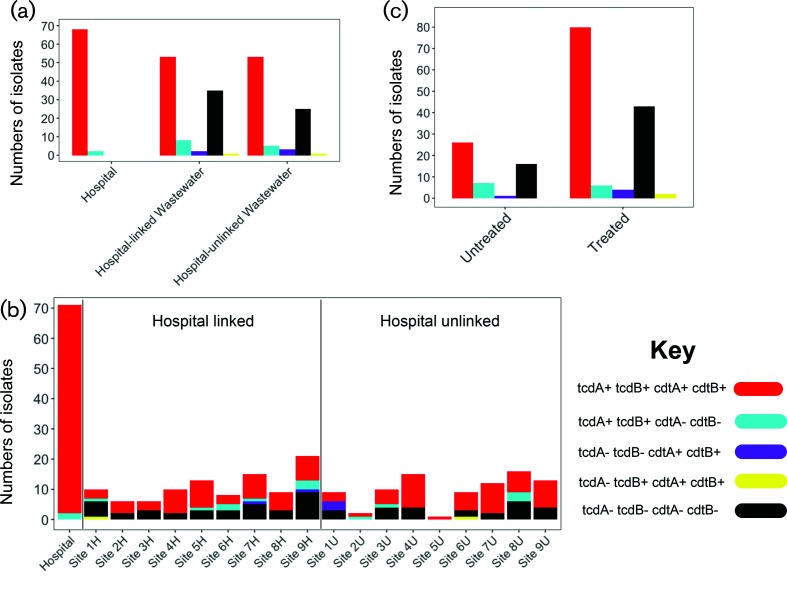
Presence of two *C. difficile* pathogenicity loci in different sub-populations. PaLoc, pathogenicity locus carrying genes encoding toxin A and toxin B. CdtLoc, pathogenicity locus carrying genes encoding the CDT binary toxin. Distribution of pathogenicity loci or toxin A and toxin B genes according to: (a) source, clinical isolates associated with *C. difficile*-associated diarrhoea, or wastewater from treatment plants that were or were not linked to hospital waste; (b) individual wastewater treatment plant; and (c) treated and untreated wastewater.

The detection of toxin A and B production by clinical isolates is integral to our diagnostic pathway (see Methods), and thus all our clinical isolates were toxigenic; 68 isolates carried both PaLoc and CdtLoc (tcdA+/tcdB+/cdtA+/cdtB+) and two isolates carried PaLoc alone (tcdA+/tcdB+/cdtA−/cdtB−). Screening of 186 wastewater isolates showed that 106 (56 %) were tcdA+/tcdB+/cdtA+/cdtB+, 13 (8 %) were tcdA+/tcdB+/cdtA−/cdtB−, five (2 %) were tcdA−/tcdB−/cdtA+/cdtB+, two (1 %) were tcdA−/tcdB+/cdtA−/cdtB− and 60 (32 %) were tcdA−/tcdB−/cdtA−/cdtB− (Fig. 3a). Two ST37 (ribotype 17) genomes contained full-length *tcdB* and mutated *tcdA* genes with a premature stop codon, which has been associated with outbreaks worldwide [35–38]. Furthermore, five isolates were tcdA−/tcdB−/cdtA+/cdtB+, all of which were ST48 isolated from three wastewater treatment plants. There was a complete absence of PaLoc in the five isolate genomes, and no replacement by an alternative element.

Toxigenic isolates were isolated from all water treatment plants, including all treated wastewater samples in these plants (Fig. 3b). The proportion of toxigenic to non-toxigenic isolates was comparable for untreated and treated wastewater, and between plants that directly received hospital waste versus those that did not (Fig. 3b, c). There was a strong correlation between phylogeny and the presence of the PaLoc and CdtLoc (Pearson correlation test: *P*<10^−3^). This is consistent with stable integration into the genome following acquisition (Figs S4 and S5). We investigated the ancestral state for the presence of the CDT locus and found that this was likely to have been present in ancestral strains (Fig. S5b) and to have been lost from the same genomic location (Fig. S5a) in non-toxigenic lineages.

Analysis of 78 additional putative virulence genes revealed that genes in the flagellar operon (implicated in adherence and linked to toxin production [39]) were over-represented in clinical isolates (Student's *t*-test *P*<10^−3^), but no other genes were over-represented in either clinical or wastewater isolates (Fig. S6).

### Distribution of antibiotic resistance determinants in *C. difficile* associated with disease and from wastewater treatment plants

Screening of all 256 isolates for genes encoding antibiotic resistance demonstrated these to be present at relatively low prevalence overall. The two most common were *ermB* encoding erythromycin resistance [33 (13 %) isolates (seven clinical, 26 wastewater)] and *tetM* encoding tetracycline resistance [18 (7 %) isolates (four clinical, 14 wastewater)] (Fig. S7). The distribution of *ermB-* and *tetM-*positive isolates across the whole collection phylogeny indicated that these genes had been gained on multiple occasions by different lineages (Fig. S7).

The mobile genetic elements (MGEs) on which *tetM* and *ermB* resided were investigated. *tetM* was identified on four distinct MGEs. The majority of *tetM-*positive isolates (13/18, 72 %) carried an accessory region (Acc1tet) that shared 99 % sequence identity with the *C. difficile* reference genome strain M120 (bases 2 171 636-2 185 712), isolated in the UK in 2007 from a human source [40]. Isolates carrying this element belonged to numerous lineages (ST11, ST29, ST35 and ST48), the last containing clinical and wastewater isolates. Two Acc1tet-positive isolates (one wastewater, one clinical; both ST11) carried an additional *tetM* accessory region (Acc2tet) which were also a close match to the genome of *C. difficile* strain MG120 (bases 416 096–485 371). A further two *tetM-*positive isolates (both ST54) carried a transposon that was highly related to Tn*6086*, and three isolates (each a different ST) carried a *tetM*-associated accessory fragment (Acc3tet), which revealed a partial match to the *Enterococcus faecium* Tn*6085* transposon. *ermB* was also associated with multiple accessory elements, although a portion of isolates (11/33, 33 %, all ST15 and including clinical and environmental isolates) carried an MGE that was closely related to the integrative mobile element Tn*6215*, identified previously in *C. difficile* strain CD80 [41]. We also identified two additional *ermB*-carrying elements that have been reported previously, namely Tn*6194* identified in *C. difficile* strain CII7 (accession HG475346) and Tn*6218* identified in *C. difficile* strain Ox42 (accession HG002387). A proportion of isolates belonging to different STs carried the *ermB* gene on a small novel accessory element of around 3.3 kb (Acc4erm). Besides antibiotic resistance genes, we identified known mutations in *gyrA* (T82I in seven clinical isolates and four wastewater isolates) and *gyrB* (S416A in seven clinical and three wastewater isolates, S366V in nine clinical isolates and one wastewater isolate, S366A in eight clinical and 11 wastewater isolates) associated with fluoroquinolone resistance [42, 43].

## Discussion

We found that *C. difficile* was extensively released into surface environmental waters, which is consistent with an earlier study across South Wales in which more than 2500 environmental water samples were tested. *C. difficile* was recovered from water samples taken from rivers, lakes and the sea [44]. Previous studies have reported that *C. difficile* survive wastewater treatment processes and have found ribotypes that were common to wastewater and clinical isolates [18–20]. However, ribotyping is known to lack sufficient genomic resolution to indicate close relatedness [11]. The use of genome sequencing allowed us to characterize and compare isolates destined for release into surface waters and isolates associated with human disease. Our study revealed multiple instances of genetic relatedness between wastewater and clinical isolates, providing strong evidence for high prevalence of *C. difficile* strains in treatment plants and overlap between these and clinically relevant strains, indicating transmission between patients and environmental waters and persistence of some genotypes in humans and the environment.

Our results suggest that circulation of *C. difficile* may include other potential reservoirs such as animal sources. For instance, the recent emergence of *C. difficile* ribotype 078 as a cause of diarrhoea in humans and its isolation from livestock farms in the Netherlands and elsewhere [33, 45] suggests that this lineage has an animal reservoir in some settings. The lineage has been increasingly identified as an emerging type with reported outbreaks in the UK [34, 46]. We isolated *C. difficile* ST11/ribotype 078 from wastewater that was genetically related to isolates from Oxford and the Netherlands, with evidence for genetic divergence over the last 20 years. This suggests inter-country as well as inter-host transmission of this important lineage.

The virulence of *C. difficile* is primarily attributed to toxin A and toxin B, located in a pathogenicity locus (PaLoc) [47], and a binary toxin encoded by the CDT locus (CdtLoc), reported to be associated with more severe disease [48]. Toxigenic *C. difficile* was extensively represented, with two-thirds of isolates positive for genes encoding toxin A, toxin B and a binary toxin encoded by the CDT locus, or genes for toxins A and B without CDT (56 and 8 %, respectively). Our findings indicate a high prevalence of toxigenic strains in wastewater and confirm that toxigenic *C. difficile*, including isolates closely related to those causing human disease, are widely dispersed across the wastewater system and are released into the environment. Human acquisition of *C. difficile* from downstream surface waters may be possible directly through recreational activities or indirectly through the use of water for irrigation of crops.

*C. difficile* carried in the human gut may act as a donor of genes encoding antibiotic resistance to other bacterial species of clinical importance. Furthermore, sewage provides a milieu in which mobile elements can be transferred within and between bacterial species. Screening of study isolates for carriage of genes encoding resistance to commonly used antibiotics suggested that these were at a relatively low prevalence. Further investigation of the genetic context of the two most frequent resistance genes (*tetM* and *ermB*) suggested that each was associated with multiple accessory elements. Taken together, our findings indicate that resistance genes were carried by diverse MGEs. However, *C. difficile* isolates belonging to the same lineage but derived from distinct sources (wastewater and clinical) carried the same antimicrobial resistance-associated MGEs. One explanation for this is sharing of MGEs related to common ancestry.

A limitation of our study was that recovery of wastewater isolates post-dated clinical isolates by 1 year. Contemporaneous data would allow us to infer directionality of transmission between the clinical and environment settings, but we propose that our findings still hold because the prevalence of *C. difficile* ribotypes was largely unchanged between 2014 and 2015 [*C. difficile* ribotyping network for England and Northern Ireland, Biennial report (2013–2015), www.gov.uk/government/uploads/system/uploads/attachment_data/file/491253/CDRN_2013-15_Report.pdf].

In conclusion, this study of the reservoirs and sources for human acquisition of *C. difficile* demonstrated extensive release of toxigenic *C. difficile* into the environment, including toxigenic isolates that were highly related to those causing clinical disease. The proportion of human disease based on the acquisition of *C. difficile* carriage from the natural environment is difficult to estimate, but the genetic similarity between isolates associated with disease and released into surface waters calls into question whether strategies are required to improve bacterial control in treated wastewater.

## Supplementary Data

2303 Supplementary File 1

## Supplementary Data

62 Supplementary File 2
