# Supplementary material for: Genomic survey of Clostridium difficile reservoirs in the East of England implicates environmental contamination of wastewater treatment plants by clinical lineages
Source: Microb Genom. 2018 Mar 2;4(3):e000162. doi: 10.1099/mgen.0.000162 (PMC5885014; doi:10.1099/mgen.0.000162)
Supplement: Supplementary File 1 [file mgen-4-162-s001.pdf]

Supplemental Material

Genomic survey of *Clostridium difficile* reservoirs in the East of England implicates environmental contamination of wastewater treatment plants by clinical lineages

---

1- Figures

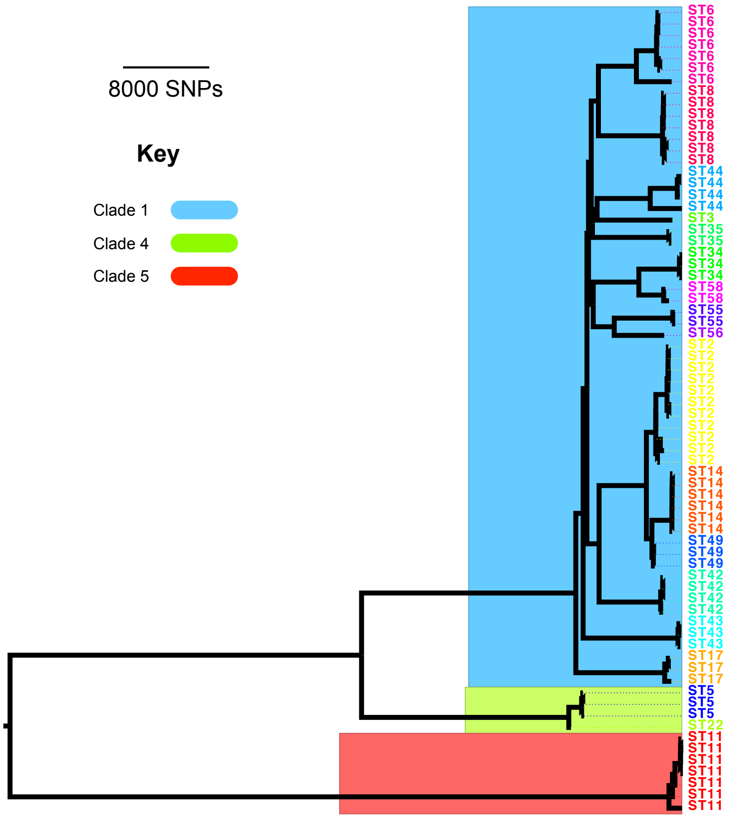

Supplementary Fig. S1 Neighbour-Joining tree of 70 clinical *C. difficile* isolates.

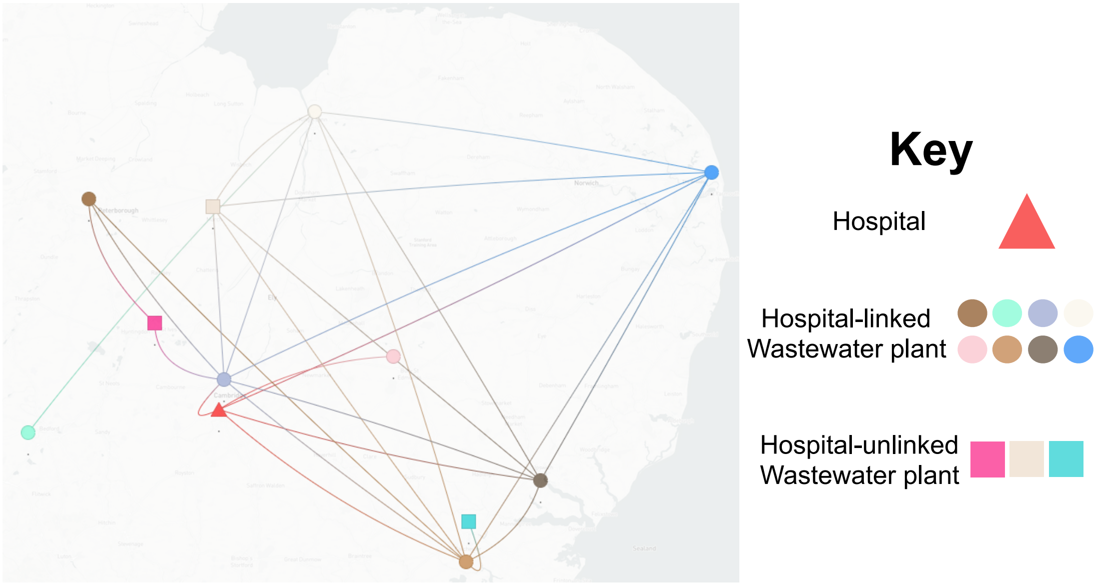

Supplementary Fig. S2 Network analysis.

Relatedness of isolates based on place of origin. Triangle, Cambridge University Hospitals NHS Foundation Trust. Wastewater treatment plants in direct receipt of hospital effluent (circle) or not in direct receipt of hospital effluent (square). The edges show isolates that were very closely related (equal to or less than 2 SNPs different on pairwise comparison of core genome).

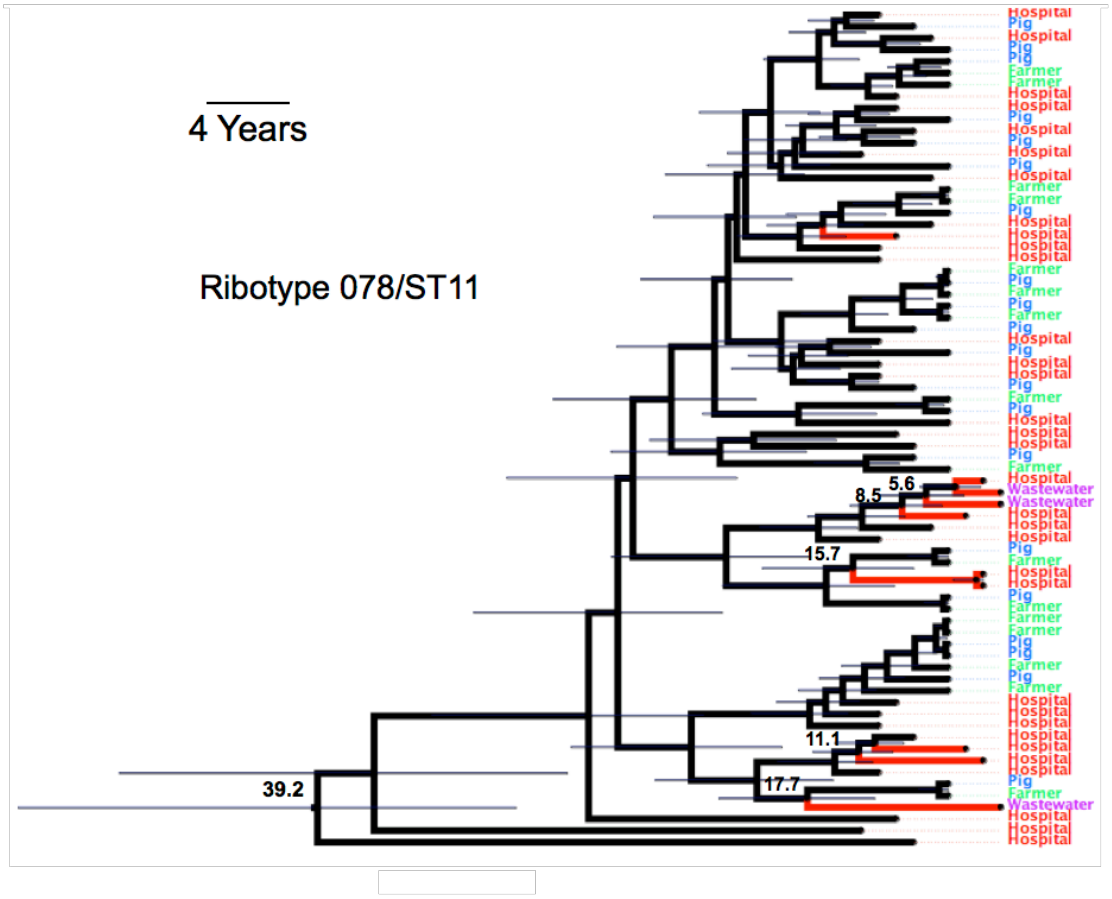

Supplementary Fig. S3 Bayesian analysis of ribotype 078/ST11 isolates.

Bayesian tree of 10 isolates from Cambridge in the UK and the Netherlands. The red and black branches correspond to Cambridge and Dutch isolates, respectively. Clinical refers to isolates associated with *C. difficile* diarrhoea. Node numbers represent node ages, and bars represent 95% height highest posterior density (HPD) intervals at 95%. 5 divergent strains from the Dutch collection are not shown in the tree.

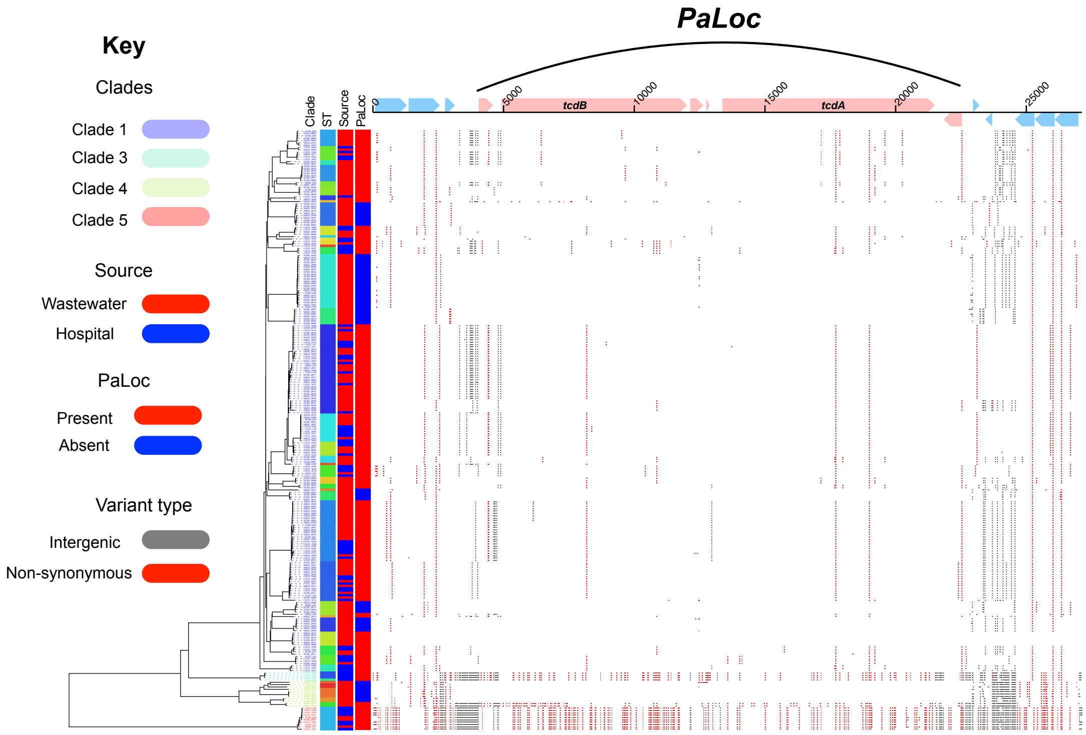

Supplementary Fig. S4 Phylogenetic distribution and genetic context of PaLoc.

Left, Neighbor-Joining tree for 256 isolate genomes (70 clinical and 186 wastewater) and presence of the PaLoc pathogenicity island based on mapping of each genome to the reference genome *C. difficile* 630. Top, genes within the PaLoc pathogenicity island (red) and upstream and downstream regions (blue). White bars correspond to absent regions. The red and black bars in the alignment represent the non-synonymous and intergenic variants. High density of non-synonymous SNPs was observed in the diverged ST11 lineage. A non-synonymous SNP in *toxA* at position 139 (C to T change) in two isolates representing the ST37 resulted in a premature stop codon and a truncated open reading frame.

A

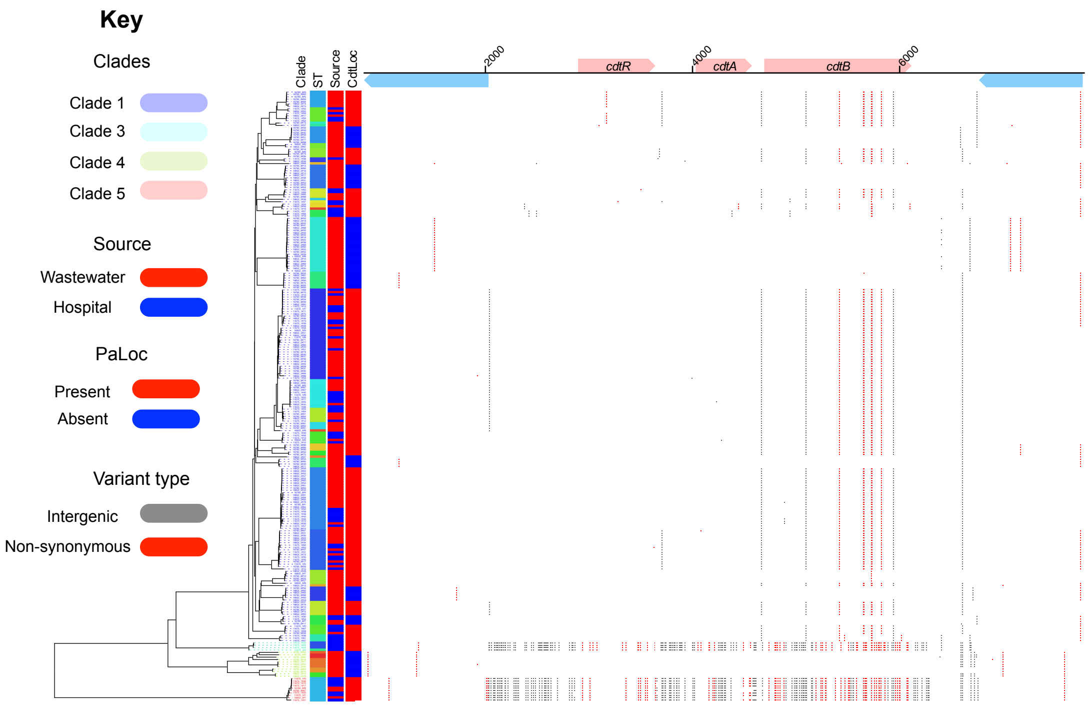

B

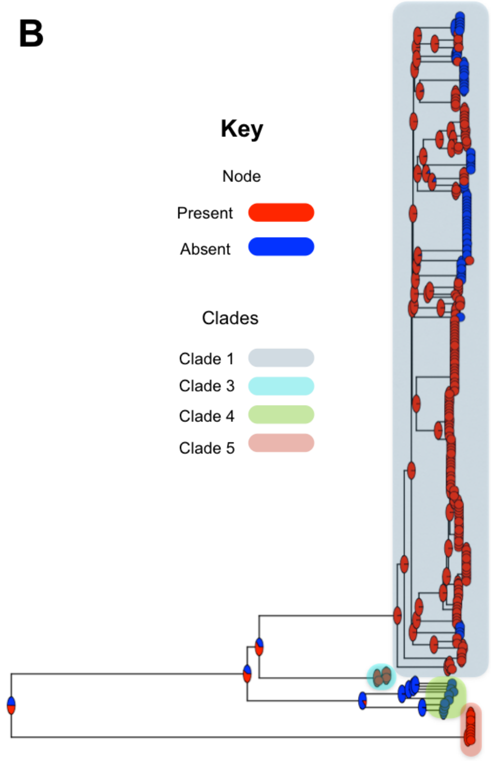

Supplementary Fig. S5 Phylogenetic distribution and genetic context of Cdt.

(a) Neighbor-Joining tree for 256 isolate genomes (70 clinical and 186 wastewater) and presence of the Cdt pathogenicity island based on mapping of each genome to the reference genome *C. difficile* 630. Top, genes within the PaLoc pathogenicity island, together with up- and downstream regions. White bars correspond to absent regions. The red and black bars in the alignment represent the non-synonymous and intergenic variants. (b) The likelihood reconstruction of the Cdt locus. Pie charts on the ancestral node denote the inferred marginal likelihood of presence (red) or absence (blue), calculated by the ancestral state reconstruction analysis.

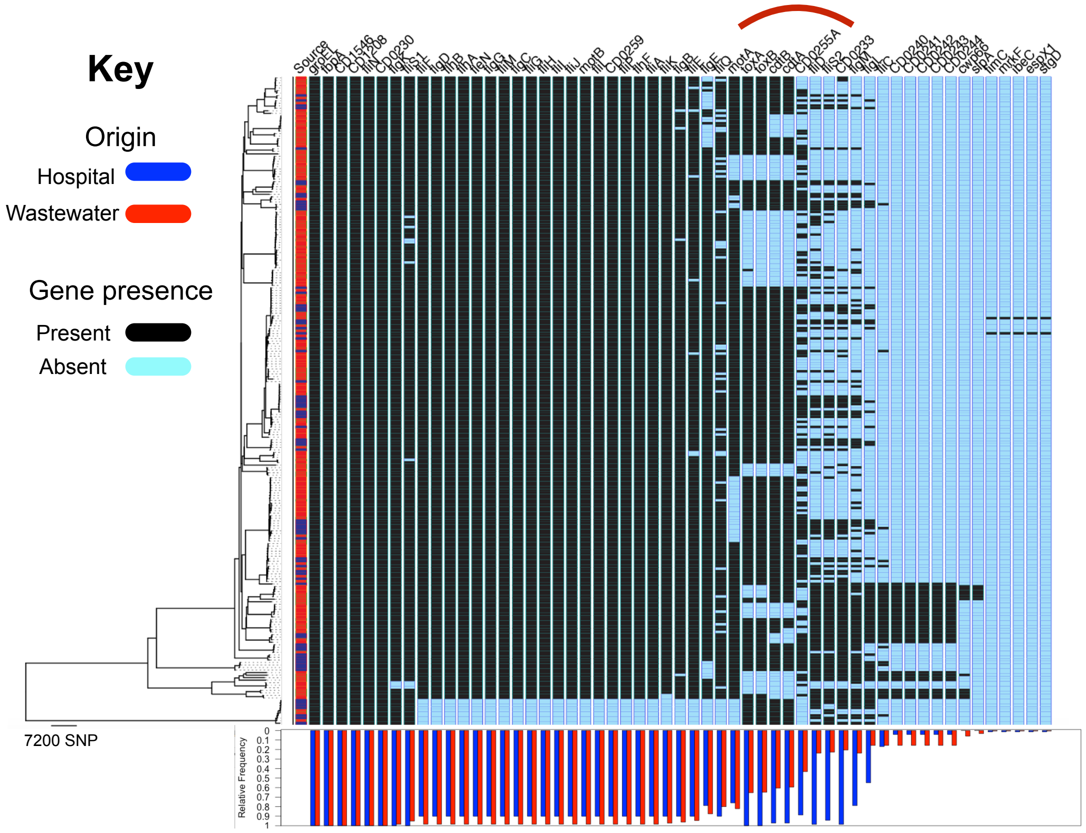

Supplementary Fig. S6 Phylogenetic distribution of putative virulence factors.

Upper image: first vertical bar relates to source of isolates: clinical (blue), wastewater plants (red). Remaining bars show the presence (black) or absence (light blue) of the gene (top labels). The gene abbreviations shown are explained in Supplementary Table S3. The first bar shows the origin of isolates for clinical (blue colour) and wastewater (red colour) isolates. The red bar (top) indicates genes that were significantly more abundant in clinical isolates compared with wastewater isolates. Lower image: relative frequency of each gene in clinical (blue colour) and wastewater (red colour) isolates. 32 genes that were present in 1 isolate in the population are not shown in this Figure.

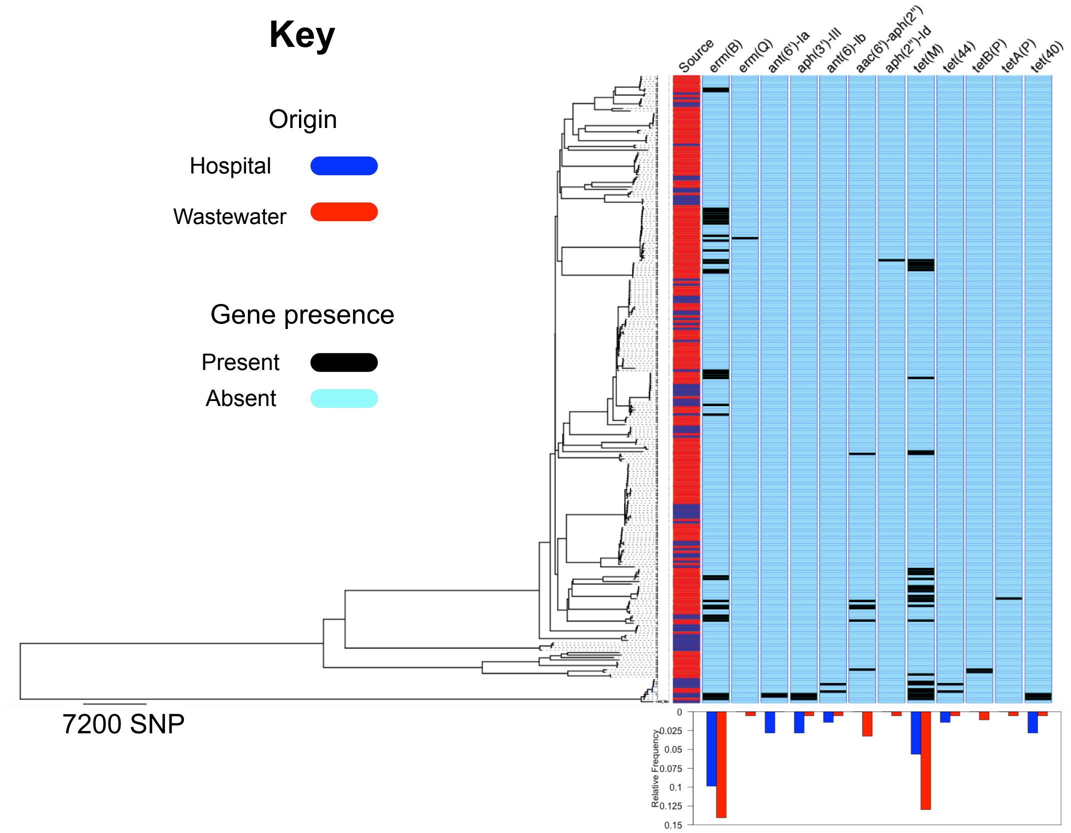

Supplementary Fig. S7 Phylogenetic distribution and genes associated with antibiotic resistance

Left, Neighbor-Joining tree for 256 isolate genomes (70 clinical and 186 wastewater). First vertical bar relates to source of isolates: clinical (blue), wastewater (red) in direct receipt of hospital waste. Remaining blocks show presence of absence of antibiotic resistance genes as detected by srst2.

2- Supplemental Tables Description

Supplementary Table S1. Epidemiological data.

Isolate and accession codes associated with data deposited in the European Nucleotide Archive (ENA) for newly sequenced isolates, or NCBI for public data used in this study.

Supplementary Table S2. Accession codes for the ST11 Dutch isolates.

Supplementary Table S3. List of virulence factors and their annotations.
